# A factor analytic investigation of the Tripartite model of affect in a clinical sample of young Australians

**DOI:** 10.1186/1471-244X-8-79

**Published:** 2008-09-18

**Authors:** Joe A Buckby, Sue M Cotton, Elizabeth M Cosgrave, Eoin J Killackey, Alison R Yung

**Affiliations:** 1 ORYGEN Youth Health Research Centre, Melbourne, Australia; 2 Department of Psychiatry, University of Melbourne, Australia; 3 Department of Psychology, University of Melbourne, Australia

## Abstract

**Background:**

The Mood and Anxiety Symptom Questionnaire (MASQ) was designed to specifically measure the Tripartite model of affect and is proposed to offer a delineation between the core components of anxiety and depression. Factor analytic data from adult clinical samples has shown mixed results; however no studies employing confirmatory factor analysis (CFA) have supported the predicted structure of distinct Depression, Anxiety and General Distress factors. The Tripartite model has not been validated in a clinical sample of older adolescents and young adults. The aim of the present study was to examine the validity of the Tripartite model using scale-level data from the MASQ and correlational and confirmatory factor analysis techniques.

**Methods:**

137 young people (M = 17.78, SD = 2.63) referred to a specialist mental health service for adolescents and young adults completed the MASQ and diagnostic interview.

**Results:**

All MASQ scales were highly inter-correlated, with the lowest correlation between the depression- and anxiety-specific scales (r = .59). This pattern of correlations was observed for all participants rating for an Axis-I disorder but not for participants without a current disorder (r = .18). Confirmatory factor analyses were conducted to evaluate the model fit of a number of solutions. The predicted Tripartite structure was not supported. A 2-factor model demonstrated superior model fit and parsimony compared to 1- or 3-factor models. These broad factors represented Depression and Anxiety and were highly correlated (r = .88).

**Conclusion:**

The present data lend support to the notion that the Tripartite model does not adequately explain the relationship between anxiety and depression in all clinical populations. Indeed, in the present study this model was found to be inappropriate for a help-seeking community sample of older adolescents and young adults.

## Background

The comorbidity of Anxiety and Mood disorders has been well established [1,2] and has led some researchers to debate whether anxiety and depression are distinct constructs or form part of a single continuum ranging from 'pure' anxiety (no depression) to 'pure' depression (no anxiety) [3,4]. The midpoint of this continuum may be marked by comorbid depressive and anxious symptoms. Indeed, anxiety and depression may share a common neuroendocrinological dysregulation [5]. In support of the continuum model is the finding that self-report scales of anxiety and depression are frequently highly correlated [6-8].

In contrast to the continuum theory is the Tripartite model of affect [9]. Central concepts to this model include Positive Affect (PA), Negative Affect (NA) and Physiological Hyperarousal (PH). PA can be considered a uniquely depression-related factor. High levels of PA relate to feelings of joy, interest and enthusiasm while low levels are represented by such constructs as fatigue and languor [9]. PH is a uniquely anxiety-related factor and is argued to capture elements of somatic tension and somatic arousal. According to the Tripartite model, the comorbidity of depression and anxiety can be explained by a shared general distress factor. This factor, characterised by high levels of NA, is defined as relating to different aspects of depression and anxiety [7]. Nervousness, tension and worry are reported as being related to anxiety, while anger, guilt and sadness are associated with depression. Both PA and NA have been argued to be relatively stable, heritable traits and largely independent of one another [10].

There are both clinical and nosological implications if the Tripartite model is found to be valid in multiple settings. For example, recent research has shown that by specifically targeting anxiety disorders with psychological treatment, significant reductions in depressive symptomatology can be achieved [11]. The authors explained this by arguing that treating the core pathology (i.e. Negative Affect), would not only impact on the target, but also on the secondary disorder (in this case, depressive symptoms). Support for the Tripartite model has also been established in recent pharmacological research [12] and by recent research that has implicated different risk factors for depression, anxiety and general distress [13]. A wide body of evidence supports the validity of the Tripartite model in distinguishing between anxiety and depression in both adult [14-17] and child/adolescent community samples [7,18-24]. Recent findings, however, have suggested a modification to the original model [25]. The anxiety-specific factor, PH, may have a heterogeneous relationship with anxiety disorders. Specifically, PH may only be related to Panic Disorder and, to a lesser extent, Generalised Anxiety Disorder. PH may be unrelated to Social Phobia and Obsessive Compulsive Disorder. Additionally, low PA has been associated with social phobia [25,26].

The Mood and Anxiety Symptom Questionnaire (MASQ) was specifically designed to measure the constructs proposed by the Tripartite model of affect [27,28]. NA is measured by three general distress scales (reflecting mixed, depressive and anxious symptoms). PA is measured by the depression-specific scale, Anhedonic Depression (AD), which incorporates both loss of interest (e.g., 'felt withdrawn from others') and high positive affect (e.g., 'felt hopeful about the future'). PH is measured by the anxious-specific scale, Anxious Arousal (AA). The validity of the MASQ was assessed across five samples: three undergraduate students; a normal adult population; and patients from a substance abuse program. Factorial validity for the MASQ has been established with a 3-factor solution consistently found to be the most appropriate fit, albeit in community rather than clinical samples with high proportions of people affected by depression and anxiety [8,16,28]. A recent clinical study, that included depressed and anxious participants, also found support for the Tripartite model in a Dutch translation of the MASQ [29]. Further psychometric support for the MASQ has been established with lower reported inter-correlations between the anxiety- and depression-specific scales (r = .25 – .49) than those reported between other self-report measures of anxiety and depression [6,28]. However, it has been argued [30] that this lower correlation may in fact represent scale unreliability rather than discriminant validity. Burns & Eidelson report that depression and anxiety (as latent constructs) correlate strongly (r at least .70) and contend that valid measures of these constructs should correlate at an equivalent level [30]. Therefore, the specific scales of the MASQ may have (comparatively) low correlations because they do not sample variance from all aspects of the construct to which they purport to measure.

The majority of reviewed studies have employed exploratory factor analysis (EFA) in their assessment of the Tripartite model. EFA provides a technique by which to delineate whether, at the item level, the factor structure, proportion of variation, and factor correlations can be replicated across various sample populations. This technique is subjective in nature and data driven [30,31]. To the best of our knowledge, only two studies have employed the more sophisticated confirmatory factor analysis with clinical samples [30,32]. Confirmatory factor analysis (CFA) is a more appropriate method for testing whether the factor structure of a covariance matrix from a novel sample can corroborate the original model [31]. Further, CFA is theory driven [30]. Burns and Eidelson [30] applied CFA to the covariance matrices of the scale-level data from Watson et al.'s substance use and student samples [27], as well as their own sample of outpatients seeking treatment for depression and anxiety. More recently, Boschen & Oei [32] employed CFA at both item-level and scale-level in a sample of Australian outpatients with mood and anxiety disorders. Neither study found support for the postulated three dimensions of the model. A 2-factor solution (marked by anxiety and depression) was found to provide a good fit across all samples by Burns and Eidelson [30]. It is worth noting however, that Burns et al. only used four of the five MASQ scales (GD: Mixed was omitted). However, none of the three tested models with scale-level data returned an adequate fit in Boschen and Oei's study [32]. This study also tested an additional four models at item-level, none of which supported the Tripartite model and which resulted in significantly poorer fit than using scale-level data. A third study has used CFA in a large sample of college students [33]. However, relatively low mean scores and a low correlation between the specific scales (r = .20) makes it difficult to directly compare this sample with those of Burns & Eidelson [30] and Boschen and Oei [32]. It may be that there is an inconsistent factor structure when comparing clinical to non-clinical samples. Interestingly, the two studies using clinical samples [30,32] have applied CFA at the scale-level rather than focusing specifically on the individual items of the MASQ. This approach allows for the theory driven testing of whether the five scales of the MASQ fit the proposed Tripartite model. This approach also has several advantages compared to item-level data analysis in terms of higher reliability, higher communality, a greater ratio to-common-to-unique factor variance, and less chance of distributional violations [34]. Further, such an approach has the benefit that fewer parameters are required to define a construct, which is a particular advantage for smaller sample sizes [34].

Therefore, two of the three published studies that have used CFA to test the Tripartite model have found support for a 2- rather than 3-factor structure of affect. This has serious implications for measures that are based upon this potentially erroneous model. The MASQ is increasingly becoming a popular self-report instrument for the dimensional assessment of anxious and depressive symptoms [35-40]. It is therefore essential that studies determine to what extent this instrument measures the domains proposed by the Tripartite model of affect in different populations to understand for which populations it may be valid. Although the Tripartite model has been extensively tested in community samples of children and younger adolescents [7,19,21,22,24,41,42] and in community and student samples, there is a dearth of information regarding its validity in clinical samples of older adolescents and young adults. Late adolescence and early adulthood is the time of peak onset of mental disorders and it has been argued that this is a life stage worthy of study in its own right [43]. Some support for the Tripartite model has been demonstrated in a clinical sample of children and adolescents (aged 7–17, mean age = 12.46), though this study only investigated two of the three tripartite constructs (NA and PA) [18].

Our recent study using a clinical population of adolescents and young adults (aged 15–24 years, mean age = 17.78) found that the MASQ did not distinguish as predicted between Mood and Anxiety disorders. Participants with depressive disorders scored higher on all MASQ scales (PA, NA and PH) than those with anxiety disorders. Participants with anxiety disorders did not score significantly higher on any MASQ scale than participants without an Axis-I disorder. We speculated that the MASQ may reflect general psychological distress in certain populations [44]. Furthermore, we have argued that the depression-specific scale demonstrated good clinical utility in its ability to distinguish between depression and anxiety, however, the anxiety-specific scale showed poor discriminant validity [45]. An additional difference between our sample and that used by Boschen and Oei is the reported correlation between the MASQ specific scales AD and AA (r = .59 vs. .45). An analysis of the difference between these correlations reveals that anxiety and depression are significantly more highly correlated in our sample of adolescent and young adult help-seekers (z = 1.96, p = .025) (see results section for detail about this calculation). We have previously found an excellent internal consistency for the AD scale, however Boschen and Oei report this scale evidenced inadequate internal consistency. There is, therefore, a clear need to replicate the findings presented by Boschen and Oei in their adult, clinical sample in order to determine whether their results generalise to younger clinical samples. It may be that the Tripartite model does not have a consistent factor structure across different samples.

Findings from correlational analyses, EFA and CFA cast some doubt about the homogeneity of the Tripartite model in clinical versus community samples and across the lifespan. The present study, which sampled older adolescents and young adults who were referred to a specialist mental health service for youth, aimed to determine whether the factor structure validated in largely non-clinical populations could be replicated in a young (aged 15–24 years) clinical sample of help-seekers. The aim of the present study is to examine the factorial validity of the Tripartite model using scale-level data from the MASQ, an instrument designed specifically to measure the postulated shared and distinct components of depression and anxiety. It was hypothesised that a factor structure previously reported in an adult clinical sample (namely, 2-factors that broadly represent depression and anxiety) would show superior fit when compared to the original Tripartite model (i.e. 3-factor).

## Methods

Ethics approval for this study was given by the local ethics board, the Melbourne Health Research and Ethics Committee (MHREC), Victoria, Australia.

### Participants

Two hundred and four people aged 15–24 years who were consecutively referred to ORYGEN Youth Health (OYH) for non-psychotic problems were invited to participate in the study. Of these, 150 consented to participate (M = 18.11, SD = 2.61). For participants aged 17 years or younger, consent was sought from a parent or legal guardian. OYH is a public mental health centre for youth in the Western and Northern region of Melbourne, Australia. Participants were eligible to take part in the study regardless of whether they were accepted into the clinical program at OYH. Exclusion criteria for this study included an inability to speak English, a known organic cause for the reason of referral to OYH, living outside the catchment area for the clinical program, having an intellectual disability or presenting with a psychotic disorder.

### Measures

#### MASQ

The Mood and Anxiety Symptom Questionnaire [27,28] is a 90-item self-report questionnaire that assesses depressive and anxious symptomatology using a 5-point Likert scale (1 = not at all, 5 = extremely). Only the 77 items retained by Watson et al. were analysed in the present study. Three scales measure General Distress: Depressive symptoms (12 items), Anxious symptoms (11 items) and Mixed symptoms (15 items). There is also an anxiety specific scale (Anxious Arousal, 17 items) and a depression-specific scale (Anhedonic Depression, 22 items). Watson et al. reported that the internal consistency of each scale was excellent with Cronbach coefficient alphas ranging from .78 to .92. Inter-correlations between MASQ scales varied widely across the five samples analysed by Watson et al. [28] with the AA: AD correlation lowest (r = .31 – .49 across samples).

#### Psychiatric Diagnosis

Axis-I psychopathology was assessed by the Structured Clinical Interview for DSM-IV (SCID-IV) [46]. Inter-rater assessments were conducted in approximately 15% of interviews to ensure agreement across raters. Kappa values for mood (.89) and anxiety (.80) diagnoses were excellent.

#### Disruptive Behaviours

The presence of Disruptive Behavioural Disorders in participants aged less than 18 was assessed by the Schedule for Affective Disorders and Schizophrenia- Children's Version (K-SADS) [47].

### Procedure

Young people were assessed by trained interviewers within two weeks of referral to OYH. Assessments included a diagnostic interview and the MASQ.

### Data Analysis

To verify the accuracy of data entry, nearly 20% of the data were re-entered and analysed for discrepancies (error rate < 0.05%). The few incorrect data points that were detected were corrected. Prior to analyses, data were screened for missing values. Thirteen cases with more than 25% of questionnaire data missing were deleted from subsequent analyses, leaving 137 participants who completed at least 75% of the items. Using the Statistical Package for the Social Sciences Version16.0 (SPSS), several missing values were replaced with the Expectation Maximisation (EM) method [48]. MASQ scales were analysed for kurtosis and skewness. Anxious Arousal scores were positively skewed (skewness = 4.63) and were corrected using a square root transformation [48]. These skew-corrected variables were used in subsequent analyses.

### Correlational Analyses

Pearson product moment correlation coefficients between MASQ scales were examined to assess the relationship between theoretically similar (e.g. AD: GD-D) and dissimilar scales (e.g. AD: AA) and therefore providing a measure of convergent and discriminant validity. Correlations around 0.10 were considered small, correlations around 0.30 moderate, and correlations 0.50 or greater large [49]. The sample was categorised according to diagnostic status and the correlations were re-examined in each of these groups to determine whether similar correlations would be observed.

### Confirmatory Factor Analysis

Confirmatory factor analyses (CFA) were performed using AMOS 7.0. Several, competing, models were investigated sequentially. In CFA, circles represent latent (not directly measured) constructs and rectangles represent directly measured variables. A single arrow denotes a predicted relationship between constructs/variables. A double headed arrow denotes predicted covariance between factors. The absence of a line between variables implies no hypothesised relationship. Maximum likelihood estimation was employed to estimate all models.

There were two potential options when analysing the latent structure of the MASQ: at item-level or at scale-level. It was determined that the present analyses would be most valid if MASQ scales were entered (i.e. Anhedonic Depression, Anxious Arousal, GD:Anxious Symptoms, GD: Depressive Symptoms, GD: Mixed Symptoms) rather than the individual 77 items. By entering the scales rather than items, we were able to maximise our ratio of participants to variables and, therefore, increasing the validity and interpretability of our subsequent results. Both previously published clinical studies that have used CFA to explore the Tripartite model have used similar methodology [30,32]. Boschen and Oei [32] also used item-level analyses but reported a substantially better fit when the scale-level analyses were conducted. The psychometric benefits of CFA on scale-level data have already been discussed [34]. Further, the focus of the current study is not on how individual MASQ items map onto the five scales, but rather how the scales fit the Tripartite theory.

### Models tested in the current analyses (see figure 1)

#### Model 1- Test of the predicted Tripartite factors

**Figure 1:**
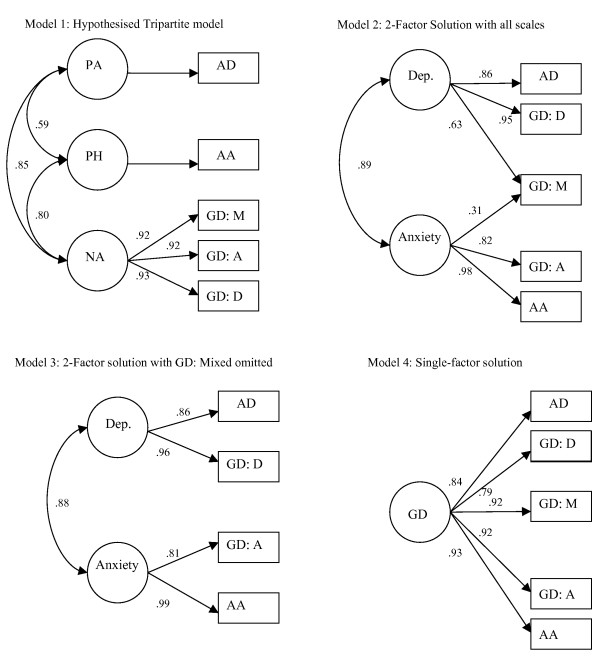
Models tested in the current analyses.

The first model was based upon the Tripartite model of affect and comprised of three latent dimensions (Positive Affect, PA; Negative Affect, NA; and Physiological Hyperarousal, PH). The depression-specific scale of the MASQ, Anhedonic Depression (MASQ: AD) was fitted to PA, the anxiety-specific scale, Anxious Arousal (MASQ: AA) was fitted to PH and the three General Distress scales (depressive symptoms, anxious symptoms and mixed symptoms) were fitted to NA (see figure 1).

#### Model 2- Test of a 2-factor model

Burns and Eidelson [15] reported a 2-factor solution best representing the MASQ data with their clinical samples. Results from a non-clinical sample also supported a 2-factor solution [18]. However, Boschen and Oei [32] found no support for the same solution. The second model therefore replicated this structure. The two latent constructs represent Depression and Anxiety. The depression scales (MASQ: AD and MASQ: GDD) were fitted to the Depression factor and the anxiety scales (MASQ: AA, MASQ: GDA) were fitted to the Anxiety factor. The mixed symptoms scale (MASQ: GDM) was fitted to both latent variables.

#### Model 3- Test of an alternative 2-factor model

The third model was a direct replication of the most parsimonious model reported by Burns and Eidelson [30]. This model was largely identical to model 2; however the mixed symptom scale was omitted from the structure. This model was justified by the high reported inter-correlations between the general distress scales that are suggestive of item redundancy.

#### Model 4- Test of a 1-factor model

The fourth model was a single-factor model that had all five MASQ scales loading onto a single General Psychological Distress latent factor.

#### Model 5- Test of the predicted Tripartite model using item-level analysis

The final model attempted to test the predicted Tripartite model. As opposed to model 1, this model attempted to fit the 77 MASQ items onto their predicted latent construct. Therefore, the MASQ: AD items were fitted to PA, the MASQ: AA items were fitted to PH, and the items from the three general distress scales were fitted to NA.

### Assessing the goodness of fit of models

A number of parameters are investigated when using CFA [48]. The first of these, the Chi Square statistic (χ^2^), tests whether the matrix of implied variances and covariances is significantly different to the matrix of empirical sample variances and covariances (i.e. the opposite of a null hypothesis). This test, with its associated degrees of freedom (df) gives a probability of significant difference. For CFA, p values greater than .05 signify that the specified model may be a feasible representation of the data it purports to portray. However, χ^2 ^is influenced by model complexity (more complex models increase the statistic and therefore increase the likelihood that the model will be accepted). Therefore, the normed Chi-Square (χ^2^/df) can also be used. Ideally, this statistic should be around 1.0 (values between 2–3 can also be considered acceptable). Values less than 1.0 indicate overfit. Standardised Root Mean-Square Residual (SRMR) and the Root Mean-Square Error of Approximation (RMSEA) were assessed. For both of these, values less than < .05 are desirable. Additionally, Goodness-of-Fit (GFI) and the Adjusted Goodness-of-Fit (AGFI) were examined. Values greater than .95 indicate satisfactory fit. A large discrepancy (> .05) between these indices indicates problems with the model. Two final indices were inspected, the Comparative Fit Index (CFI) and the rho2. Both these indices require a value greater than .95 for a well fitting model. rho 2 can exceed 1.0, however larger values indicate over-specification of the model. Finally, the Akaike Information Criterion (AIC) was inspected. This is a measure of model parsimony. There are no defined 'acceptable' levels for this criterion; however, in a group of models, the model with the lowest AIC value is to be considered the most parsimoniously fitting model.

## Results

### Sample Characteristics

The mean age of participants was 17.78 years (*SD *= 2.63, range = 15 – 24). The sample consisted of 61% females (*n *= 84). There were no significant differences in age between males and females (*t *(135) = 0.04, p = 0.97). Demographic data for non-consenters could not be collected. The majority (80.2%, *n *= 109) of the sample reached diagnostic threshold for an Axis-I disorder. There were no significant age or gender differences between those with a disorder and those without. Mood and Anxiety disorders were most common in this sample. Fifty seven participants (37.7%) had an Anxiety disorder and 64 (47.1%) rated for a Mood Disorder. The most common Anxiety disorders in this sample were Social and Specific Phobias (15.4% each) Panic Disorder (8.8%) and Post Traumatic Stress Disorder (8.1%). Obsessive Compulsive Disorder (6.6%), Generalised Anxiety Disorder (5.1%) and Agoraphobia (2.2%) were less common. Substance Abuse/Dependence and Disruptive Behaviour Disorders were next most commonly diagnosed (22.1% and 20.0% respectively). There was no significant difference in the proportion of males (40%) and females (52%) with a Mood Disorder (p = .17). More females (49%) than males (30%) rated for an Anxiety Disorder (p = .03).

### Descriptive Statistics

Means and standard deviations of the MASQ scales are presented in Table 1. Descriptive statistics from six other studies are also detailed for comparison. There were no significant differences between males and females for any of the MASQ scales in the present study. The mean scores in the present study were generally higher than previously reported in non-clinical samples, but were lower than reported in an inpatient sample.

**Table 1:** Means and Standard Deviations of MASQ Scores Across Studies- Analysed by Gender

		**Current ** **Study**	Reidy & Keogh (1997)	Watson et al. (1995a)	Watson et al. (1995a)	Nitsche et al. (2001)	Ruth & Mehrotra (2001)	Clark et al. (1998)	Geisser et al. (2006)	
	Sample:	**Outpatient**	student	substance use patient	student	student	patient	inpatient	pain clinic patients	community pain patients
	Mean Age:	**17.78**	27.54	39.3	-	-	28	41.12	46.3	53.8
			Scale Means (SD)							

Male	GD: M	**41.23 (13.67)**	33.56 (12.30)	34.90 (12.30)	34.50 (9.00)	-	-	-	-	-
	GD: A	**23.17 (8.43)**	19.63 (7.70)	21.60 (7.50)	22.30 (6.40)	-	-	-	-	-
	GD: D	**30.58 (11.98)**	22.06 (10.20)	28.00 (10.00)	24.50 (8.70)	-	-	-	-	-
	AA	**32.78 (13.24)**	26.91 (11.10)	28.30 (10.40)	27.80 (9.40)	-	-	-	-	-
	AD	**74.15 (15.92)**	54.72 (16.50)	65.50 (14.80)	55.60 (13.40)	-	-	-	-	-

Female	GD: M	**46.09 (14.61)**	32.33 (10.60)	-	35.20 (9.20)	-	-	-	-	-
	GD: A	**26.44 (9.81)**	19.17 (6.80)	-	23.60 (6.30)	-	-	-	-	-
	GD: D	**35.05 (13.17)**	21.69 (9.40)	-	25.80 (8.80)	-	-	-	-	-
	AA	**34.91 (13.48)**	24.23 (8.80)	-	27.10 (8.20)	-	-	-	-	-
	AD	**76.86 (18.34)**	56.52 (14.70)	-	54.20 (13.90)	-	-	-	-	-

Total	GD: M	**44.20 (14.40)**	-	-	-	36.76 (9.21)	33.27 (13.47)	49.41 (7.18)	37.7 (11.9)	32.1 (10.2)
	GD: A	**25.17 (9.40)**	-	-	-	22.88 (6.29)	21.82 (9.06)	31.53 (6.78)	22.9 (7.3)	19.7 (6.4)
	GD: D	**33.31 (12.86)**	-	-	-	27.24 (9.01)	26.12 (11.79)	45.21 (5.86)	27.1 (10.9)	22.4 (8.6)
	AA	**34.08 (13.38)**	-	-	-	27.60 (8.29)	29.63 (13.96)	34.12 (10.25)	31.2 (10.1)	26.9 (7.5)
	AD	**75.81 (17.43)**	-	-	-	57.39 (13.73)	64.98 (16.93)	87.53 (9.48)	66.9 (16.2)	57.3 (15.6)

*Note*. Dash indicates data not reported by that study. GD: M = General Distress: Mixed symptoms; GD: A = General Distress: Anxious symptoms; GD: D = General Distress: Depressive symptoms; AA = Anxious Arousal; AD = Anhedonic Depression.

The reported internal consistency for each MASQ scale, as measured by Cronbach's coefficient alpha, were excellent (α = .88 – .93) (see diagonal of Table 2).

**Table 2:** Internal consistency and correlations between MASQ scales

	GD: M	GD: A	GD: D	AA	AD
GD: M	(.92)				
GD: A	.86**	(.88)			
GD: D	.86**	.83**	(.93)		
AA	.74**	.80**	.69**	(.91)	
AD	.79**	.75**	.82**	.59**	(.93)

*Note*. Scale reliabilities are shown on diagonal in parentheses.

GD: M = General Distress: Mixed symptoms; GD: A = General Distress: Anxious symptoms; GD: D = General Distress: Depressive symptoms; AA = Anxious Arousal; AD = Anhedonic Depression;

** denotes significant at p < .001.

### Correlational Analyses

For the total sample, correlations between the scales ranged from 0.59 (for AA:AD) to 0.86 (for GD:A and GD:D) (see Table 2). The sample was split into the following diagnostic groups: Mood Disorder Only (no Anxiety Disorder, n = 29); Anxiety Disorder Only (no Mood Disorder, n = 22); Comorbid Anxiety-Depression (n = 35); Other DSM-IV disorder (n = 23); and No DSM-IV disorder (n = 27). Correlations between the two disorder-specific scales, AA and AD, were inspected for each group. This correlation was high in all groups with Axis-I disorders with the highest AA:AD correlation in Anxiety Only (r = .62), followed by Comorbid (.60), Other (.48) and Mood Only (.46) (p < .001 for all). In contrast, AA:AD were only weakly correlated in those subjects without an Axis I disorder (*r *= 0.18, *p *= 0.36). The AA:AD correlation for participants with any Axis-I disorder (r = .59, p < .001, n = 109) was compared to the no diagnosis group (n = 27). An independent samples t-test of the correlational coefficients was conducted [50], showing a significantly higher correlation in the composite diagnosis group (*z *= 4.04, *p *< .001).

### Confirmatory Factor Analysis of MASQ items

Using CFA, five models were devised and sequentially analysed to determine which demonstrated the best fit. The hypothesised model (model 1, see figure 1 and Table 3) demonstrated acceptable fit indices. However, this model demonstrated a high normed Chi Square, indicating poor fit to the data. Models two through four were next inspected. Model 2 (representing a 2-factor solution with all five MASQ scales) showed a significant increase in parsimony when compared to the predicted Tripartite structure. The normed Chi Square was in the acceptable range, had good fit indices and a lower AIC. Model 3 (2-factor solution that did not include the GD: Mixed symptoms scale) was judged to be the best fitting model as evidenced by acceptable normed Chi Square, good fit indices and the lowest AIC of any tested model. The highest loading item on each factor was the general symptom (i.e. GD: D, GD: A) rather than specific symptom (i.e. AD, AA) scale. Across models 1–3, high correlations between the depression and anxiety latent constructs were found (r = .85 – .89), indicating near collinearity between these supposedly distinct constructs. However, despite this very high correlation, reducing the model to a single factor solution (model 4) significantly compromised the parsimony of the model. Model 4 had a high normed Chi Square, mixed fit indices, and the second highest AIC of any model. Formally testing the possibility that depression and anxiety actually represent a single, general psychological distress, construct (i.e. by setting the correlation between the latent constructs to 1.0 resulted in a significantly poorer model than the single factor model (model not presented as a figure), χ^2 ^(2) = 163.21, p < .001.

**Table 3:** Fit indices for the 4 models assessed through Confirmatory Factor Analysis

		**Model Fit Indices**									
		
	**Model No**.	χ^2^	df	χ^2^/df	p	GFI/AGFI	CFI	rho2	RMSEA	SRMR	AIC
1	Predicted Model (3-factor)	21.12	4	5.28	< .001	.94/.78	.98	.94	.18	.025	43.12
2	2-Factor Model (all scales)	3.10	3	1.03	.38	.99/.96	1.00	1.00	.014	.012	27.08
**3**	**2-Factor Model (without GD:M)**	**1.30**	**1**	**1.3**	**.26**	**.99/.95**	**1.00**	**.99**	**.047**	**.009**	**19.30**
4	Single Factor Model	31.0	5	6.2	< .001	.91/.73	.96	.92	.20	.033	51.00
5	Predicted Model (item-level)	5492.4	2846	1.93	< .001	.50/.49	.67	.66	.08	.09	5806.41

*Note*: Model 3 (in bold) was judged to be the best-fitting model. The normed Chi square was close to 1, p vale was non-significant, fit indices were within acceptable limits and had the lowest AIC value of any of the tested models.

χ^2 ^= Chi Square, df = degrees of freedom, GFI = Goodness of Fit Index, AGFI = Adjusted Goodness of Fit Index, CFI = Comparative Fit Index, RMSEA = Root Mean-Square Error of Approximation, SRMR = Standardised root Mean-square Residual, AIC = Akaike Information Criterion.

Finally, using item-level (as opposed to scale level in the first four models) analysis, fitting the MASQ items to their predicted latent construct resulted in a significant decrease in parsimony compared to all models tested at scale level. Model 5 had a high normed Chi square, poor indices and a very high AIC.

## Discussion and conclusion

The present results did not support the predicted structure of the Tripartite model in a clinical sample of older adolescents and young adults. A confirmatory factor analysis (CFA) was conducted to determine whether a 3-factor model (as predicted by the Tripartite model) would best represent the latent structure of affect in a young, clinical sample. In CFA, variables are forced onto factors and the resulting competing solutions can be directly compared. The hypothesised model (model 1, see figure 1) demonstrated acceptable fit indices but was not judged to show good parsimony to the data. In a series of subsequent analyses, 1-, and 2-factor models were individually assessed (see data analysis section [above] for a description of each model and Table 3 for fit indices).

In contrast to the predicted 3-factor (tripartite) structure, a 2-factor solution was analysed next. The latent constructs in these solutions broadly represented Depression and Anxiety. The first of these (model 2) resulted in a good model fit and proved to be more parsimonious than the predicted model. However, the correlation between the two broad constructs (depression and anxiety) was very high (r = .89), indicating near multi-collinearity (whereby two constructs are so highly related that they can be considered a single construct). It was hypothesised that the mixed symptoms subscale of the MASQ may be causing this high correlation due to item overlap across factors. The MASQ: GDM scale is comprised of items that relate to both anxiety and depression and was highly correlated with both GD: D and GD: A (see Table 2). This scale was therefore removed for model 3 (replicating the most parsimonious model presented by Burns and Eidelson [30]), resulting in an increase in goodness-of-fit and an increase in parsimony (as evidenced by the lowest AIC). However, Depression and Anxiety remained highly correlated at a factorial level (r = .88).

Finally, a single-factor solution was assessed (model 4). This model resulted in mixed findings for the fit indices and significantly higher AIC scores. It was therefore determined that a single-factor model was a poor fit to the present data with young help-seekers.

Previously, Cole et al. investigated the structure of the Tripartite model in a community sample of children (mean ages for the cohorts = 8.9 and 11.9 years) [51]. They reported two broad factors emerging (depression and anxiety) but also reported that these factors were so highly correlated that they were essentially indistinguishable. This highly correlated 2-factor model has also been replicated in large samples of substance abusers (r = .81) and adult outpatients (r = .75). The present results lend support to the notion that Depression and Anxiety may exist as broad, but highly related, constructs in young people in the emerging phase of psychiatric disorders. There was little support in the present findings for the predicted mixed depression-anxiety factor (NA).

Previous research has implicated that the items in the depression-specific scale of the MASQ may in fact represent two distinct clusters of items ('Loss of Interest' and 'High Positive Affect') [10,27,29]. Preliminary analyses with the current sample showed a similar pattern of results to those obtained elsewhere (data from these exploratory analyses are not presented in the present manuscript but are available from the corresponding author upon request). This possibility (that the depression scale is best represented by two distinct subscales) was assessed in revisions to models 1–4 (models not presented). However, splitting the AD items resulted in a reduction of parsimony and it was therefore determined that the depression-specific scale was best represented as a single construct. This finding provides further evidence for the assertion that a likely method effect underpins the distinction between these sets of positively- and negatively-valanced items [29].

An alternative interpretation of the present findings may offer support for a dimensional representation of mental disorders. One could argue that there are no separate anxiety and depression factors in the present sample. Rather, there is only a single 'general psychological distress' factor. Although there was a reduction in model parsimony when considering a single factor solution (model 4), the high correlation between Depression and Anxiety in the model with the best fit (model 3, r = .88), would support this argument. Inspection of present eigenvalues reveals that the first factor (eigenvalue = 3.25) accounted for 38.7% of the explained variance. The next factor had a noticeably lower eigenvalue (0.45). This strong, general factor which accounts for the majority of the explained variance has also been identified in previous studies with different populations [10,27], indicating the current sample is not anomalous.

The mean scores for the current study are higher than those typically reported in the literature but lower than reported with inpatients (see Table 1), indicating the present sample was more highly distressed than those generally studied. It is possible that in the present clinical sample of highly distressed young people, a ceiling effect may have occurred, making anxiety and depression somewhat indistinguishable and limiting the utility of the Tripartite model for this population. Previous research with older adult clinical samples has also cautioned that the Tripartite model may require revision in different populations [15].

Results from the correlational analyses with the MASQ scales offer further support for this proposition. The specific scales AD and AA were strongly correlated for the sample as a whole and in each examined diagnostic category (r = .48 – .62). These correlations were statistically higher than for those participants who did not have a diagnosis (r = .18). Given that the MASQ was specifically designed to measure the postulated constructs of the Tripartite Model, these results indicate that this model may not differentiate between Anxiety and Depression as well in young, clinical samples as in older non-clinical samples, which further supports some recent research. We have previously reported that the anxiety-specific scale of the MASQ does not distinguish between participants with and without Anxiety disorders [44]. The depression scale however does have good clinical utility [45]. The high inter-correlation between AA and AD in participants with disorders is similar to that reported with Indian adult outpatients (r = .49) [52]. High inter-correlations between Tripartite dimensions (r = .71 – .84) in urban African-American youth have also been reported, leading the authors to argue that anxiety and depression may not homogenously differentiate [20]. Indeed, recent revisions to the Tripartite Model have argued that the anxiety-specific factor PH may only relate to Panic Disorder and to a lesser extent GAD [53]. While distinct anxious and depressive syndromes may be found in relatively healthy samples of students and adults, this may not be the case in clinical samples with high levels of depressive and anxious symptomatology. The present sample may best be considered as a group of young people with high levels of general psychological distress. Help-seekers are a group who are more severely ill and more likely to have comorbid disorders when compared to people with psychiatric disorders who do not seek help [54]. Thus, the present sample of help-seekers may not be representative of the wider population. A recent study with a clinical adult sample found no support for the Tripartite model across any of the tested models [32]. The present results, using a sample that was similar in composition but markedly different in age yielded very different results, further supporting the possibility that the Tripartite model may not be homogenously represented across different settings. For many within the present sample, their non-specific symptoms may not yet have clearly differentiated into depressive and anxious disorders. It is therefore not surprising that their scores on specific measures of anxiety and depression are highly correlated, as they are likely to be reporting high rates of comorbid symptoms. Indeed, of the 86 participants with either an Anxiety of Mood Disorder, nearly half (41%) were comorbid for both disorders. This sample therefore is clearly different to that examined in previous studies [8,16,28,32].

The sample size in the present study was less than generally recommended for factor analytic investigations [48]. Comparisons between the present factor structures identified in preliminary analyses (see footnote 2) and that reported elsewhere reveals similar trends across different studies, further indicating that the present sample size is large enough for the results to be generalisable to other populations. In addition, the decision was made to analyse the data at scale-level rather than item-level in order to maximise the ratio of cases to variables. The use of scale-level or parcelled scores (averaging or summing scores across items) has the advantages of ameliorating the effects of non-normally distributed item-level data and is particularly useful when the factor structure is known [55]. Greater reliability of the scale-level data, greater communality, and a larger ratio of common-to-unique factor variance are also some psychometric benefits of this approach [34]. Scale-level analysis is also appropriate when the items comprising the scale score represent a unidimensional construct [34,55,56]. However, problems can arise in the use of this technique when the items parcelled are multidimensional or when there is inconsistent information about the factor structure underlying the items [55], as may be the case with the MASQ scales. There has been controversy to whether scale-level analysis improves the fit of the model [34]. However, we found that testing the Tripartite model at item level (model 5) resulted in a significant decrease in model parsimony, further supporting the use of current methodology.

In conclusion, the present findings from a clinical sample of older adolescents and young adults did not support the hypothesised factor structure of the tripartite model as measured by the MASQ (a self-report questionnaire specifically designed to test the latent constructs of this model). Our findings indicate that the MASQ, and hence the Tripartite model, may require further revision in clinical populations. Despite Watson et al.'s [28] conceptualisation that specific components of Anxiety (Physiological Hyperarousal) and Depression (Positive Affect) can be separated from shared symptoms (Negative Affect), the present findings indicate that in young, clinical samples, only two broad (but highly correlated) constructs exist. This strong relationship (r = .88 at a factorial level and r = .59 for the specific scales of the MASQ) may be related to NA's merging into the Depression and Anxiety factors. Despite this strong relationship, the present data did not support a single-factor modelling of Depression and Anxiety. These results require replication with larger samples of help-seeking adolescents and young adults.

## Competing interests

The authors declare that they have no competing interests.

## Authors' contributions

JB collected the data, performed the statistical analyses, formulated the research question and wrote this manuscript. SC provided assistance with statistical analyses and helped to draft the manuscript. AY conceived the study, participated in its design, helped draft the manuscript and obtained the funding. EC participated in the design of the study and co-ordination and helped to draft the manuscript. EK participated in the design of the study and co-ordination. All authors read and approved the final manuscript.

## Pre-publication history

The pre-publication history for this paper can be accessed here:


